# Characteristics of slow progression to diabetes in multiple islet autoantibody-positive individuals from five longitudinal cohorts: the SNAIL study

**DOI:** 10.1007/s00125-018-4591-5

**Published:** 2018-03-12

**Authors:** Anna E. Long, Isabel V. Wilson, Dorothy J. Becker, Ingrid M. Libman, Vincent C. Arena, F. Susan Wong, Andrea K. Steck, Marian J. Rewers, Liping Yu, Peter Achenbach, Rosaura Casas, Johnny Ludvigsson, Alistair J. K. Williams, Kathleen M. Gillespie

**Affiliations:** 1 Translational Health Sciences, Bristol Medical School, University of Bristol, Level 2, Learning and Research, Southmead Hospital, Bristol, BS10 5NB UK; 2 Division of Endocrinology and Diabetes, Children’s Hospital of University of Pittsburgh Medical Center, Pittsburgh, PA USA; 3 Division of Infection and Immunity, Cardiff School of Medicine, Cardiff University, Heath Park, Cardiff, UK; 4 Barbara Davis Center for Childhood Diabetes, University of Colorado School of Medicine, Aurora, CO USA; 5 Institute of Diabetes Research, Helmholtz Zentrum München and Forschergruppe Diabetes, Klinikum rechts der Isar, Technische Universität München, München, Germany; 6 Division of Pediatrics, Department of Clinical and Experimental Medicine, Faculty of Medicine and Health Sciences, Linköping University, Linköping, Sweden

**Keywords:** HLA class II, Islet autoantibodies, Slow progression, Type 1 diabetes

## Abstract

**Aims/hypothesis:**

Multiple islet autoimmunity increases risk of diabetes, but not all individuals positive for two or more islet autoantibodies progress to disease within a decade. Major islet autoantibodies recognise insulin (IAA), GAD (GADA), islet antigen-2 (IA-2A) and zinc transporter 8 (ZnT8A). Here we describe the baseline characteristics of a unique cohort of ‘slow progressors’ (*n* = 132) who were positive for multiple islet autoantibodies (IAA, GADA, IA-2A or ZnT8A) but did not progress to diabetes within 10 years.

**Methods:**

Individuals were identified from five studies (BABYDIAB, Germany; Diabetes Autoimmunity Study in the Young [DAISY], USA; All Babies in Southeast Sweden [ABIS], Sweden; Bart’s Oxford Family Study [BOX], UK and the Pittsburgh Family Study, USA). Multiple islet autoantibody characteristics were determined using harmonised assays where possible. HLA class II risk was compared between slow progressors and rapid progressors (*n* = 348 diagnosed <5 years old from BOX) using the *χ*^2^ test.

**Results:**

In the first available samples with detectable multiple antibodies, the most frequent autoantibodies were GADA (92%), followed by ZnT8A (62%), IAA (59%) and IA-2A (41%). High risk HLA class II genotypes were less frequent in slow (28%) than rapid progressors (42%, *p* = 0.011), but only two slow progressors carried the protective HLA DQ6 allele.

**Conclusion:**

No distinguishing characteristics of slow progressors at first detection of multiple antibodies have yet been identified. Continued investigation of these individuals may provide insights into slow progression that will inform future efforts to slow or prevent progression to clinical diabetes.

**Electronic supplementary material:**

The online version of this article (10.1007/s00125-018-4591-5) contains peer-reviewed but unedited supplementary material, which is available to authorised users.

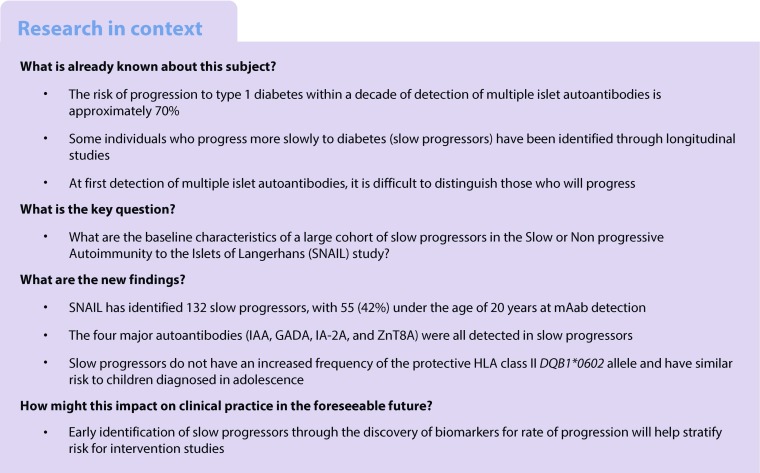



## Introduction

Immune-mediated destruction of pancreatic beta cells progresses at different speeds in different individuals. Prospective birth cohort studies show that autoantibodies to insulin (IAA), GAD (GADA), islet antigen-2 (IA-2A), and zinc transporter 8 (ZnT8A) can be detected in children at risk of type 1 diabetes from 6 months of age with a peak in seroconversion between 2 and 3 years [1]. Over 90% of individuals diagnosed in childhood have at least one of these autoantibodies [2]. Presence of multiple islet autoantibodies (mAabs) is normally associated with a disease risk of 70% within 10 years [2–5], but many individuals present with clinical symptoms later in adult life [1].

Conceptually, disease pathogenesis can be divided into three stages: development of autoimmunity as indicated by the presence of mAabs, defined recently as Stage 1; progression from autoimmunity to glucose intolerance (Stage 2); and finally to overt disease (Stage 3) [6]. These stages appear to be controlled by different genetic mechanisms, with different HLA alleles affecting seroconversion and/or progression to disease [7, 8]. Positivity for and/or higher levels of IAA, IA-2A, IA-2βA and ZnT8A have also been associated with more rapid development of hyperglycaemia [9–11].

The Slow or Non progressive Autoimmunity to the Islets of Langerhans (SNAIL) study focuses on slow progression to diabetes by studying a large international collection of individuals who develop islet autoimmunity (defined by presence of mAabs) but do not develop disease for at least 10 years. The characteristics of humoral autoimmunity in the earliest mAab-positive sample and the HLA class II profile in this unique ‘slow progressor’ cohort are described here. SNAIL participants derive from BABYDIAB [12], the Diabetes Autoimmunity Study in the Young (DAISY) [13], All Babies in Southeast Sweden (ABIS) [14], the Bart’s Oxford (BOX) Family Study [15] and the Pittsburgh Family Study [5]. These studies all investigated the natural history of diabetes and the contribution of islet autoantibodies to disease risk. Three studies (BABYDIAB, DAISY and ABIS) followed children from birth (with blood samples taken at 9 months or 1 year), while two studies enrolled first degree relatives of individuals with diabetes throughout life. Slow progressors may provide new insights that inform disease prevention strategies in those at risk of type 1 diabetes and help to identify biomarkers associated with slow progression.

## Methods

### Participants

Slow progressors were characterised by remaining diabetes-free for at least 10 years after mAabs (two or more of IAA, GADA, IA-2A or ZnT8A) were first detected. As a control group, genetic data were available for 2075 individuals diagnosed with type 1 diabetes under the age of 21 years and enrolled in the BOX study. Of these, 348 were ‘rapid progressors’ being diagnosed at less than 5 years of age and 1217 were diagnosed over 10 years of age. All individuals were participants in existing studies approved by local ethical review boards. Written informed consent was obtained from participants and/or their parents/legal guardians.

### Participating study cohorts

SNAIL participants were recruited from several natural history studies, which have been described in detail elsewhere (see electronic supplementary material [ESM] Table 1). Brief descriptions of the studies are given below.

#### BABYDIAB, Germany

BABYDIAB is a longitudinal study examining the natural history of islet autoimmunity and type 1 diabetes in 1650 children born to a mother or father with type 1 diabetes [12]. Recruitment began in 1989 and ended in 2000.

#### DAISY, USA

DAISY has followed two cohorts of young children at increased risk of type 1 diabetes (total *n* = 2547); a cohort of relatives of individuals with type 1 diabetes (siblings and offspring) and a newborn high genetic risk general population cohort from the Denver area [13, 16, 17]. Recruitment began in 1993 and ended in 2004, and follow-up is ongoing.

#### ABIS, Sweden

All mothers that gave birth in southeast Sweden between October 1997 and October 1999 were invited to participate [14]. In total 17,055 children were recruited (78.6% of all births), of whom 7394 provided at least two samples for autoantibody analysis.

#### The BOX family study, UK

BOX is a longitudinal study examining risk factors for type 1 diabetes in siblings or parents (2774 families) of a proband diagnosed under the age of 21 [15]. Autoantibodies were tested in at least one sample from 5881 relatives, diabetes-free at the time of testing. Of these, 284 have had a diabetes (any type) diagnosis. Recruitment began in 1985 and is ongoing. All participants in SNAIL were recruited before 2001.

#### The Pittsburgh Family Study, USA

More than 10,000 first-degree relatives of children and adolescents with type 1 diabetes (<19 years old) were recruited from the Children’s Hospital of Pittsburgh registry and followed up from 1979 until 2015 [18]. Relatives were excluded at screening if an OGTT or random glucose level was >7.8 mmol/l [19]. All relatives were screened using islet cell antigen (ICA) testing. Relatives confirmed positive for ICA were tested for IAA, GADA and IA-2A, this included 1484 relatives recruited between 1979 and 1984.

### Follow-up

Participants were followed for development of disease through: (1) annual or semi-annual written or telephone contact to ascertain self-reported, clinician diagnosed diabetes; (2) in ABIS, diagnosis was ascertained from the national registration of all individuals with type 1 diabetes [11]; (3) for BABYDIAB and willing DAISY participants, oral glucose tolerance tests were performed at least annually and/or HbA_1c_ monitored [2].

### Autoantibody assays

Islet autoantibodies (GADA, IA-2A, IAA and ZnT8A) were tested in each parent study by radiobinding assays as previously described [2, 3, 11, 16, 19–21] and an overview of the testing strategy for each study is summarised in ESM Methods. Positivity was defined using a threshold determined by each laboratory and where multiple samples were available, persistent autoimmunity was confirmed. Assays for GADA and IA-2A were calibrated against the WHO standard in international workshops and where samples were available, the presence of mAabs within individuals was confirmed with harmonised assays for GADA and IA-2A [20]. Where harmonised assays failed to confirm mAab, those individuals were excluded.

### Genetics

The HLA class II genotype DRB1*03-DQB1*02 (DR3-DQ2)/DRB1*04-DQB1*0302 (DR4-DQ8) (or DQB1*02 [DQ2]/DQB1*0302 [DQ8] if DR typing was not available) was considered high risk, presence of one or two copies of DR3-DQ2 or DR4-DQ8 was considered to confer intermediate risk, while all other genotypes or haplotypes containing DQB1*0602 were considered low risk. HLA typing for BOX and ABIS were carried out at the University of Bristol [22]; other studies provided their own data [5, 13, 23].

### Data analysis

Comparisons between antibody prevalence and haplotype frequencies across studies and genotype frequencies between SNAIL participants and rapid progressors were made using *χ*^2^ tests.

## Results

Overall, 132 participants were identified who remained diabetes-free for more than 10 years after mAabs were first detected (Table 1). After this 10-year period, participants remained under follow-up (median 4 years, IQR 2–9 years). During follow-up in SNAIL, 42 slow progressors were diagnosed with diabetes, but 90 remained diabetes-free based on lack of self-reported disease, absence from a diabetes case registry or lack of metabolic abnormalities.

**Table 1:** Description of participants in the SNAIL study

Characteristic	Overall	BABY DIAB	DAISY	ABIS	BOX	Pittsburgh
*n*	132	22	30	11	36	33
Age at first antibody test; Median (IQR)	7 (1–18)	1	1 (1–2)	1	18 (13–38)	18 (11–33)^a^
Age at mAab^+^ sample; Median (IQR)	10 (5–20)	5 (2–5)	7 (4–10)	5	18 (13–38)	18 (11–37)
Male; *n* (%)	69 (54)	16 (73)	17 (57)	7 (64)	15 (42)	17 (52)
Years of follow-up since mAab^+^ detection; Median (IQR)	14 (12–19)	13 (11–14)	12 (1–13)	13 (13–14)	17 (14–24)	20 (13–26)
Diabetes-free at follow-up; *n* (%)	90 (68)	12 (55)	23 (77)	10 (100)	22 (61)	23 (70)
Genetic data available	121	22	30	10	36	23

^a^Earliest sample available

IQR, interquartile range; mAab^+^, multiple autoantibody-positive

### Islet autoantibody frequency in slow progressors differs between study cohorts

**Fig. 1:**
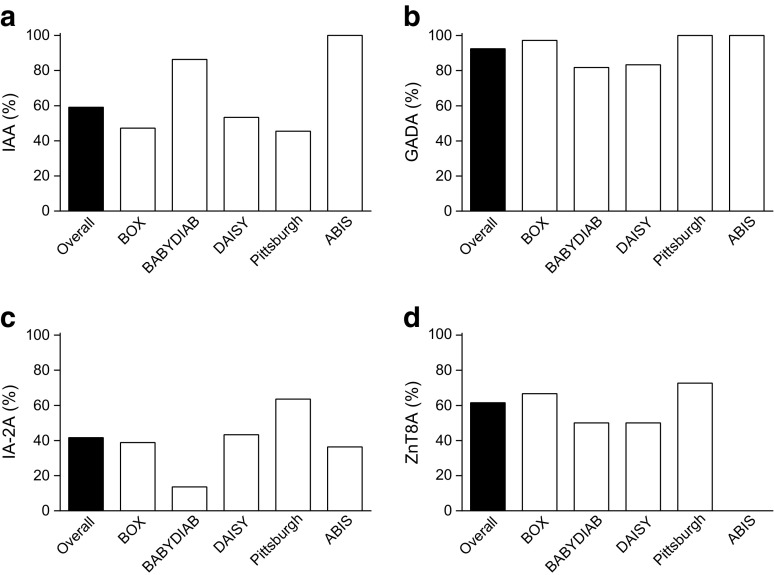
Proportion of slow progressors positive for (**a**) IAA, (**b**) GADA, (**c**) IA-2A and (**d**) ZnT8A at the first available mAab-positive sample differed between the cohorts (*p*=0.001, *p*=0.018, *p*=0.007 and *p*=0.183, for each antibody, respectively). Black bars show overall percentage, white bars show percentage for slow progressors from each study. The ABIS participants were not tested for ZnT8A in their first mAab-positive sample

The most frequent autoantibodies in the first mAab-positive samples were GADA (92%), followed by ZnT8A (62%), IAA (59%) and IA-2A (41%) (Fig. 1a–d). Of 117 individuals tested for all four antibodies (Table 2), nine (8%) had four antibodies, with the most common combination of three antibodies being GADA+IA-2A+ZnT8A (in 22 individuals, 19%) and of two antibodies being IAA+GADA (in 31 individuals, 26%). The frequency of IAA, GADA and IA-2A, but not ZnT8A, differed between cohorts (*p* = 0.001, *p* = 0.018, *p* = 0.007 and *p* = 0.183, respectively, Fig. 1). In ABIS, of ten slow progressors tested at age 11 years for ZnT8A, five were positive.

**Table 2:** Combinations of autoantibodies at first mAab-positive visit for 117 slow progressors tested for IAA, GADA, IA-2A and ZnT8A

Autoantibody combination	Number of individuals (%)
IAA+GADA+IA-2A+ZnT8A	9 (8)
IAA+GADA+IA-2A	6 (5)
IAA+GADA+ZnT8A	11 (9)
IAA+IA-2A+ZnT8A	2 (2)
GADA+IA-2A+ZnT8A	22 (19)
IAA+GADA	31 (26)
IAA+IA-2A	0
IAA+ZnT8A	6 (5)
GADA+IA-2A	8 (7)
GADA+ZnT8A	20 (17)
IA-2A+ZnT8A	2 (2)

### Slow progressors carry less HLA risk than individuals diagnosed in childhood

**Fig. 2:**
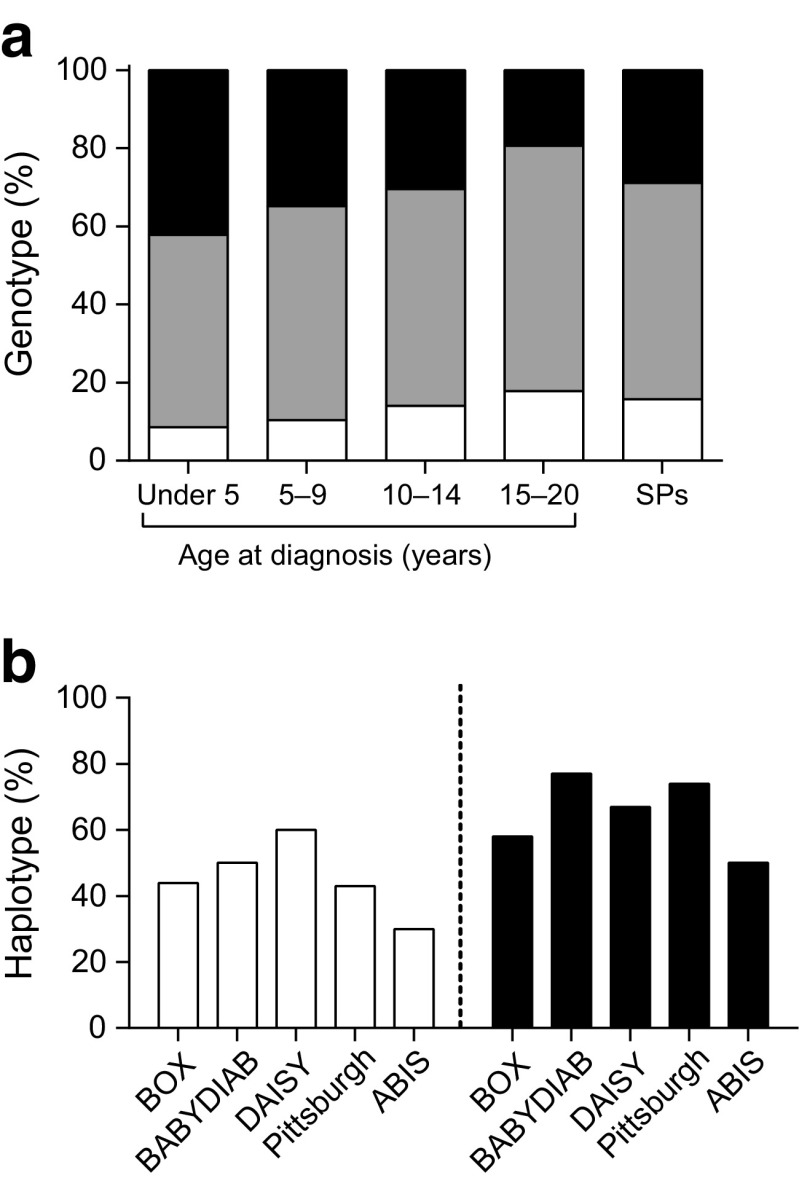
HLA risks for (**a**) proportion of HLA class II high risk (black), intermediate risk (grey) or low risk (white) genotypes in BOX probands according to age at diagnosis (*n*=2075, including 348 rapid progressors diagnosed under 5 years of age) and slow progressors (SPs, *n*=121, *p*=0.011 for HLA class II risk in slow vs rapid progressors); and (**b**) proportion of participants carrying HLA class II DQ2 (white) or DQ8 (black) haplotypes in BOX (*n*=36), BABYDIAB (*n*=22), DAISY (*n*=30), Pittsburgh study (*n*=21) and ABIS (*n*=10)

High risk HLA class II (DQ2/DQ8) was less frequent (28% vs 42%) while intermediate risk genotypes were more common (55% vs 49%) in the 121 slow progressors with HLA class II data available, than in the 348 children from BOX diagnosed under 5 years of age, who were designated rapid progressors (*p* = 0.011, Fig. 2a). Genetic risk of the slow progressors was similar to that of the 1217 BOX participants diagnosed over 10 years of age (DQ2/DQ8, 26%). A similar proportion, two of 121 (1.7%) slow progressors, carried the protective DQB1*0602 allele compared with six (1.7%) rapid progressors. HLA class II genetic risk varied between cohorts, but the frequency of DQ2 and DQ8 alleles was not significantly different (Fig. 2b).

## Discussion

Here we describe a unique group of 132 slow progressors with mAab positivity who remained diabetes-free for at least 10 years while participating in five international natural history studies. Most remained diabetes-free at last contact but could be expected to develop diabetes in the future. Indeed, 32% developed diabetes during 4 years of SNAIL follow-up. Diabetes diagnosis was self-reported for the BOX and Pittsburgh studies. This analysis brought together data from several well-characterised long running studies which will enable further analysis of these rare individuals. Most study participants were first-degree relatives of people with type 1 diabetes but also included individuals selected for HLA risk and from the general population.

All five ‘parent’ studies are longitudinal in nature; serum samples were collected and assayed over decades. Slow progressors were initially identified from historic antibody assay screening results. To allow comparison of data, where possible, the initial antibody-positive sample and/or subsequent samples were retested using standardised assays to confirm autoantibody status and these results are reported here.

The definition of slow progressor was the same for all cohorts included in this analysis, although the parent studies had different structures. The BOX and Pittsburgh family studies recruited relatives of individuals with diabetes at different ages and the age of seroconversion is often unknown. In contrast, BABYDIAB, ABIS and most DAISY participants have been studied from birth and provide valuable evidence that even individuals who develop antibodies early in life can remain diabetes-free for many years. Diabetes was defined by clinical disease, but metabolic abnormalities may appear before overt disease. All participants from BABYDIAB were normoglycaemic, while 1 of 10 DAISY participants tested, and 3 of 11 BOX participants tested recently, have elevated HbA_1c_. In BABYDIAB the slow progressors described here represent 25% of the children who became mAab-positive in the study [24]. Together these cohorts cover all stages in the natural history of islet autoimmunity; however, individuals with a family history of disease are better represented. The only cohorts included that are representative of the general population are the ABIS study where individuals with no family history were recruited and DAISY where some were recruited from the general population after genetic screening.

All SNAIL participants had mAabs, but the proportion of individuals with each autoantibody varied between study cohorts. The first antibodies to be detected in about two-thirds of young children in BABYDIAB were IAA and their loss was associated with delayed progression [25]. In the SNAIL cohorts, IAA were more common in the first mAab-positive samples from BABYDIAB and ABIS children, but half of BOX and Pittsburgh family study participants were also IAA-positive in their first sample, despite most being tested first as adults. Autoantibodies to GAD are the first islet autoantibody detected in about a third of children who develop diabetes but are also prevalent in adult onset disease [21, 26]. In SNAIL participants, GADA were common in all cohorts but more frequent in older individuals. Antibodies that recognise IA-2 and ZnT8 develop later in disease pathogenesis and are associated with progression [11]; however, ZnT8A were the second most frequent antibodies found in slow progressors. In contrast, IA-2A were less common and BABYDIAB participants had a particularly low prevalence of IA-2A (14%). During follow-up, however, 18 of 22 (82%) BABYDIAB SNAIL participants seroconverted to IA-2A positivity, indicating that antigen spreading continued despite slower progression (data not shown).

The slow progressors in SNAIL have a lower prevalence of DQ2/DQ8 compared with well-characterised rapid progressors diagnosed under the age of five from BOX but a similar genetic risk profile to individuals diagnosed in adolescence. In contrast, BABYDIAB showed that children who develop islet autoantibodies early in life have similar high HLA class II risk, independent of whether they progress to diabetes [24]. One reason for this discrepancy may be that the average age of detection of islet autoantibodies in SNAIL participants was during adolescence. Later development of autoantibodies is associated with lower HLA class II risk as these genes are known to influence antibody development, whereas HLA class I genes have a more dominant role in progression from autoimmunity to disease [7, 8]. The frequency of DQ2 and DQ8 did not differ between cohorts despite differences in participant age between the studies, but this may have been due to a lack of statistical power for comparisons between cohorts. The different structures, ages or ethnicities of the SNAIL cohorts should be considered in interpretation of our analyses. HLA class II risk in children diagnosed under 5 years old is likely to be similar in Western populations, i.e. 36–42% have the highest HLA risk in the type 1 diabetes genetics consortium, DAISY and BOX studies [27]. In addition, over 95% of participants in BABYDIAB, ABIS, BOX and Pittsburgh were of white European origin. For older participants, the age of multiple antibody seroconversion is not known, limiting the interpretation of HLA class II data. Those who seroconverted at a younger age however appear to have similar HLA class II regardless of future progression rate.

On the basis of their mAab positivity, slow progressors are at high risk of developing type 1 diabetes. Despite having a lower genetic risk of disease than rapid progressors, almost 30% of slow progressors carry the highest HLA class II risk haplotype, highlighting the importance of other factors in influencing progression to clinical disease. Continued study of SNAIL participants will focus on characteristics of the immune response, including antibody characteristics, and investigate whether these individuals develop the disease slowly or not at all because of immune regulation.

## Electronic supplementary material

ESM (PDF 96.5 kb)

## Data Availability

Requests for data sharing would need to be referred to the studies in question. More information can be provided by the corresponding author if required.

## Abbreviations

ABIS: All Babies in Southeast Sweden
BOX: Bart’s Oxford Family Study
DAISY: Diabetes Autoimmunity Study in the Young
GADA: Autoantibodies to GAD
IA-2A: Autoantibodies to islet antigen-2
IAA: Autoantibodies to insulin
ICA: Islet cell antigen
mAab: Multiple islet autoantibody
SNAIL: Slow or Non progressive Autoimmunity to the Islets of Langerhans
ZnT8A: Autoantibodies to zinc transporter 8
