# Supplementary material for: Characteristics of slow progression to diabetes in multiple islet autoantibody-positive individuals from five longitudinal cohorts: the SNAIL study
Source: Diabetologia. 2018 Mar 12;61(6):1484–90. doi: 10.1007/s00125-018-4591-5 (PMC6449004; doi:10.1007/s00125-018-4591-5)
Supplement: Supplementary file 1 — (PDF 96.5 kb) [file 125_2018_4591_MOESM1_ESM.pdf]

ESM Methods: Overview of historical and harmonized islet autoantibody testing strategy for each ‘parent’ study.

BABYDIAB; All serum samples from BABYDIAB were tested previously for GADA, IA-2A, and IAA in Munich [1, 2]. The last available sample from these participants was tested for ZnT8A and where an individual was positive, all previous available samples were also tested [3]. Later samples were tested using the harmonized assays for GADA and IA-2A [4]. Slow progressors were mAab positive in at least two sequential samples.

DAISY; Measurement of islet autoantibodies to insulin, GAD65, IA-2 and ZnT8 was performed in the Clinical Immunology Laboratory at the Barbara Davis Center (BDC) using radio-immunoassays as described previously [5, 6]. The initial assay for GADA and IA-2A was a combined dual label radioassay [5]. Since January 2010, GADA and IA-2A have been measured using the National Institute of Diabetes and Digestive and Kidney Diseases harmonised assays [4]. All available samples from children who were ever found positive for any of the above autoantibodies or who developed type 1 diabetes were tested for ZnT8A as previously described [7]. Slow progressors were antibody positive in at least two sequential samples and all were mAab positive on at least one occasion.

ABIS; All antibody testing was carried out by the pediatric research laboratory, Linköping University , Sweden. Samples taken at age 1 and 2.5-3 years were tested on EDTA whole blood for GADA and IA-2A only [8], subsequent serum samples taken at age five years and up were tested for IAA in addition to GADA and IA-2A [9]. All slow progressors were identified through measurement of GADA, IA-2A, and IAA. After 11 years of age, ZnT8A were also tested [10]. Data were provided for 24 ABIS participants with mAabs who had not progressed to diabetes before 11 years of age. Of these, 11 were identified as Slow Progressors.

BOX; The first available serum samples from relatives in BOX and the last sample for those with a follow-up of at least 4 years were previously tested for GADA and IA-2A at the University of Bristol [11]. Insulin autoantibodies were also tested in all relatives under the age of 10 years. To identify multiple antibody positive individuals IAA and ZnT8A were tested in all IA-2A and/or GADA positive relatives [12, 13]. Multiple antibody positive samples from potential slow progressors were retested with harmonised assays for GADA and IA-2A [4], as well as for IAA and ZnT8A when serum was available, these results were used to define Slow Progressors. Where multiple samples were available, only individuals with persistent mAabs were included.

Pittsburgh family study; All serum samples from relatives in the Pittsburgh study were tested historically for IAA, GADA and ICA 512 (IA-2A) at the University of Pittsburgh from 1979 to 1984 and 2004 to 2011. In the intervening years the biochemical antibodies were tested if screening for ICA was positive or the participants developed diabetes of any type. Potential slow progressors were initially selected from those previously found positive for IAA, GADA, IA-2A, or high levels of ICA [14]. Multiple antibody positive samples from these potential slow progressors were retested with harmonised assays for IAA, GADA, IA-2A and ZnT8A [4, 6, 15] at the Clinical Immunology Laboratory at the BDC. Slow progressors were defined based on the results of harmonised assays.

References for online supplemental material 2.

1. Ziegler AG, Hummel M, Schenker M, Bonifacio E (1999) Autoantibody appearance and risk for development of childhood diabetes in offspring of parents with type 1 diabetes. The 2-year analysis of the German BABYDIAB study. Diabetes 48:460-468
2. Naserke HE, Bonifacio E, Ziegler AG (1998) Immunoglobulin G insulin autoantibodies in BABYDIAB offspring appear postnatally: Sensitive early detection using a Protein A/G-based radiobinding assay. Journal of Clinical Endocrinology and Metabolism 84:1239-1243
3. Achenbach P, Lampasona V, Landherr U et al (2009) Autoantibodies to zinc transporter 8 and SLC30A8 genotype stratify type 1 diabetes risk. Diabetologia 52:1881-8

4. Bonifacio E, Yu L, Williams AJK et al (2010) Harmonization of glutamic acid decarboxylase and islet antigen-2 autoantibody assays for national institute of diabetes and digestive and kidney diseases consortia. J Clin Endocrinol Metab 95:3360-7
5. Yu L, Rewers M, Gianani R et al (1996) Antiislet autoantibodies usually develop sequentially rather than simultaneously. Journal of Clinical Endocrinology and Metabolism 81:4264-4267
6. Yu L, Robles DT, Abiru N et al (2000) Early expression of antiinsulin autoantibodies of humans and the NOD mouse: evidence for early determination of subsequent diabetes. Proc Natl Acad Sci U S A 97:1701-6
7. Wenzlau JM, Juhl K, Yu L et al (2007) The cation efflux transporter ZnT8 (Slc30A8) is a major autoantigen in human type 1 diabetes. Proc Natl Acad Sci U S A 104:17040-5
8. Wahlberg J, Fredriksson J, Nikolic E, Vaarala O, Ludvigsson J, Group AB-S (2005) Environmental factors related to the induction of beta-cell autoantibodies in 1-yr-old healthy children. Pediatr Diabetes 6:199-205
9. Holmberg H, Vaarala O, Sadauskaite-Kuehne V, Ilonen J, Padaiga Z, Ludvigsson J (2006) Higher prevalence of autoantibodies to insulin and GAD65 in Swedish compared to Lithuanian children with type 1 diabetes. Diabetes Res Clin Pract 72:308-14
10. Vaziri-Sani F, Delli AJ, Elding-Larsson H et al (2011) A novel triple mix radiobinding assay for the three ZnT8 (ZnT8-RWQ) autoantibody variants in children with newly diagnosed diabetes. J Immunol Methods 371:25-37
11. Bingley PJ, Bonifacio E, Williams AJ, Genovese S, Bottazzo GF, Gale EA (1997) Prediction of IDDM in the general population: strategies based on combinations of autoantibody markers. Diabetes 46:1701-10
12. Williams AJ, Bingley PJ, Bonifacio E, Palmer JP, Gale EA (1997) A novel micro-assay for insulin autoantibodies. J Autoimmun 10:473-8
13. Long AE, Gooneratne AT, Rokni S, Williams AJK, Bingley PJ (2012) The role of autoantibodies to zinc transporter 8 in prediction of type 1 diabetes in relatives: lessons from the European Nicotinamide Diabetes Intervention Trial (ENDIT) cohort. J Clin Endocrinol Metab 97:632-7
14. Pietropaolo M, Yu S, Libman IM et al (2005) Cytoplasmic islet cell antibodies remain valuable in defining risk of progression to type 1 diabetes in subjects with other islet autoantibodies. Pediatr Diabetes 6:184-92
15. Wenzlau JM, Liu Y, Yu L et al (2008) A common nonsynonymous single nucleotide polymorphism in the SLC30A8 gene determines ZnT8 autoantibody specificity in type 1 diabetes. Diabetes 57:2693-7

ESM Table 1

Study title	BABYDIAB	The Diabetes Autoimmunity Study in the Young (DAISY)		All Babies In Southeast Sweden (ABIS)	Bart's-Oxford Family Study (BOX)	Pittsburgh Family Study
**Location**	Germany	Denver, Colorado, USA		South-East Sweden	Former Oxfordshire Health Authority, UK	Pittsburgh, Pennsylvania, USA
**Recruitment period**	1989 to 2000	1993 to 2004		1997 to 1999	1985 to present; all Slow progressors recruited before 2002	1979 to 1984
**Type**	Longitudinal from birth (9 months)	Longitudinal from recruitment (< 7 years)	Longitudinal from birth (9 months)	Longitudinal from birth (1 year)	Longitudinal from recruitment	Longitudinal from recruitment
**Participants**	Children born to a mother or father with diabetes	Siblings or offspring (< 7 years old) of an individual with type 1 diabetes	HLA-DR/DQ genotypes identified through screening of over 31,000 newborns at St. Joseph Hospital in Denver, Colorado	All babies due to be born between October 1997 and October 1999	Siblings and parents of probands diagnosed under the age of 21 years living in the region.	First degree relatives of probands diagnosed (1950 onwards) under the age of 19 in the Children's Hospital of Pittsburgh registry. Normoglycaemia at recruitment
**Genetic selection**	None	None	All children with DR3/4, DQB1*0302, DR3/3, and DR4/4, DQB1*0302 and a sample of those with DR4/DRx, DQB1*0302, or DR3/DRx (where DRx≠DR3 or DR4) were invited to participate in DAISY None	None	None	None
**Participants (n)**	1650	2547		17055; n=7394 providing at least two samples for aab analysis	1865 families before 2002	>10,000 First degree relatives
**Ethnicity**	97% Caucasian	72% non-Hispanic white		>95 % Caucasian	>95% Caucasian	96 % White
**Islet autoantibodies tested**	IAA, GADA, IA-2A, ZnT8A	IAA, GADA, IA-2A, ZnT8A		GADA and IA-2A in all samples, IAA at 5yrs and above, ZnT8A at 11 years and above	IAA, GADA, IA-2A, ZnT8A	IAA, GADA, IA-2A (in ICA+) ZnT8A (in slow progressors)
**Islet autoantibody testing schedule**	9 months, and at 2, 5, 8, 11, 14, 17 and 20 years of age. Children with a positive autoantibody finding, were asked to provide a sample for confirmation of autoantibody status within 6 months and provide samples yearly.	From recruitment <7 years. Then as with HLA screened population.	9 months, 15 months, 24 months and annually thereafter; autoantibody positive children are followed and tested for islet autoantibodies, random BG and HbA1c every 3-6 months.	1, 2.5-3, 5-6 and 8 and 11 years of age, In participants with multiple antibodies and then every 6 months for 30 month. Up to 5-6 years in association with well baby clinics; After this by mail.	From recruitment; up to 6 monthly	From recruitment; up to 6 monthly
**OGTT assessment**	Yearly in autoantibody positive	6 monthly where participants are willing. 3 monthly HbA1C.		None	Not routine	At recruitment
**Diabetes definition**	Yearly: ADA criteria >2 abnormal OGTTs or following the family reporting diabetes diagnosed by a clinician	2 yearly: ADA criteria >2 abnormal OGTT or following the family reporting diabetes diagnosed by a clinician		Swedish national incidence and quality registry for childhood diabetes SWEDIABKIDS.	Yearly: Family reporting of diabetes diagnosed by a clinician	2 Yearly: Family reporting of insulin treated diabetes diagnosed by a clinician
**Key references for study description**	Ziegler, 1999, Diabetes	Norris, 1996, JAMA	Rewers, 1996, Diabetologia	Nygren, 2015, Diabetologia; Ludvigsson, 2001, Pediatric Diabetes	Bingley, 1989, BMJ	Pietropaolo, 2005, Pediatric Diabetes; Morran, 2010, Endocrinology.

ESM Table 1: Summary of the protocols for SNAIL study cohorts.
